# Insights from metagenomics on microbial biosynthesis of vitamins B and K_2_ in chicken gut microbiota

**DOI:** 10.3389/fvets.2025.1646825

**Published:** 2025-08-14

**Authors:** Hai-Long Yu, Xin-Wen Hou, Ji-Xin Zhao, Guo-Hua Liu, Jin-Xin Meng, Yong-Jie Wei, Yanan Cai, Jian Liu, Kai-Meng Shang, Hong-Bo Ni, Rui Liu, He Ma, Fu-Long Nan, Xiao-Xuan Zhang, Bei-Ni Chen, Xing Yang

**Affiliations:** ^1^Department of Medical Microbiology and Immunology, School of Basic Medicine, Dali University, Dali, Yunnan Province, China; ^2^College of Veterinary Medicine, Jilin Agricultural University, Changchun, Jilin Province, China; ^3^College of Veterinary Medicine, Qingdao Agricultural University, Qingdao, Shandong Province, China; ^4^Research Center for Parasites and Vectors, College of Veterinary Medicine, Hunan Agricultural University, Changsha, China; ^5^Department of Veterinary Medicine, College of Animal Sciences, Zhejiang University, Hangzhou, Zhejiang, China

**Keywords:** vitamins B and K_2_, chicken gut microbiome, metagenomic analysis, cobalamin, pathogen infection

## Abstract

**Introduction:**

The chicken gut microbiome plays a pivotal role in nutrient absorption and overall health, contributing to the biosynthesis of essential vitamins. However, the biosynthesis of vitamins B and K_2_ by the whole gut microbiome, as well as their abundances across different gut regions, remains largely unknown.

**Methods:**

We employed both metagenomic sequencing and culture-based techniques, collecting a total of 25,825 genomes (25,764 metagenome-assembled genomes and 61 isolated genomes). After quality assessment and average nucleotide identity (ANI), 13,734 genomes were retained for downstream analysis.

**Results:**

Whole-genome clustering analysis identified 2,675 species-level genome bins (SGBs), predominantly from the phyla *Bacillota, Bacteroidota, Pseudomonadota,* and *Actinomycetota*. A gene catalog comprising 9.69 million genes revealed that 195,517 genes are involved in the biosynthesis of vitamins B and K_2_, exhibiting significant regional variation. The large intestine exhibited greater species richness and evenness compared to the small intestine. From the 13,734 genomes, we discovered 3,063 high-quality ones capable of synthesizing at least one vitamin. Genomic analysis revealed that a mere 8.2% of genomes were capable of producing five or more vitamins, while almost half were limited to synthesizing just one. Comparative genomics of cobalamin (B_12_) biosynthesis highlighted the predominance of the anaerobic pathway. Additionally, changes in microbial abundance were observed, such as increased abundance of the genera *Phocaeicola* and *Faecalibacterium* during bacterial infections, and *Limisoma* during parasitic infections.

**Discussion:**

This study provides detailed metagenomic insights into the capacity of chicken gut microbiome for vitamins B and K_2_ biosynthesis, revealing significant regional and taxonomic variations. These results suggest a collaborative microbial effort in vitamin biosynthesis, with potential implications for optimizing poultry health and nutrition through targeted microbial interventions.

## 1 Introduction

The gut microbiota is a complex and dynamic ecosystem that contributes to various physiological functions (1–4), including nutrient absorption, immune regulation, and pathogen defense (5, 6). Its ability to synthesize essential vitamins B and K_2_, such as thiamine (B_1_), riboflavin (B_2_), niacin (B_3_), pantothenate (B_5_), pyridoxine (B_6_), biotin (B_7_), folate (B_9_), cobalamin (B_12_), and menaquinone (K_2_), is particularly crucial for the overall health and growth of chickens (7, 8). Vitamins, acting as coenzymes (9), are indispensable in metabolic pathways including DNA synthesis, energy production, and amino acid metabolism, all of which are critical for cellular function and growth (10–13). For instance, pantothenate, a member of the vitamin B family, serves as a prosthetic group of coenzyme A and an integral component of acyl carrier proteins (ACPs). It plays a pivotal role in the metabolism of carbohydrates, lipids, and proteins within the organism (14). Deficiencies in vitamins B and K_2_ can adversely affect reproductive and production performance in chickens. For instance, riboflavin deficiency can lead to decreased body weight, egg weight, and egg production, which may subsequently cause embryo mortality (15). Similarly, shortages in pyridoxine can induce poor growth in chickens, loss of appetite, nervous signs, and chondrodystrophy (16). The exogenous supplementation of vitamins has long been a common practice to meet the nutritional needs of poultry (17). Given the high cost of supplements, improving the vitamin biosynthesis capabilities of gut microbiota and reducing dependence on external vitamin supplementation are essential to decrease chicken production costs. Therefore, a comprehensive understanding of vitamins B and K_2_ biosynthesis by the chicken gut microbiome is essential for devising strategies to regulate the production of these vitamins in the chicken digestive system.

Recently, significant progress has been made in uncovering the mechanisms of vitamin biosynthesis, especially within the gut microbiota of humans and ruminants (9, 18–20). These studies have revealed how microbes synthesize various vitamins through complex metabolic pathways, thereby influencing the nutritional status and health of the host. For instance, thiamine can be synthesized through distinct pathways involving the independent formation of thiazole and pyrimidine components. In contrast to the *NMNAT* branch, the *NaMNAT* branch in niacin biosynthesis exhibits greater prevalence within the gastrointestinal tract (GIT) microbiome of ruminants (20). Notably, cobalamin has the most complex biosynthesis pathway among all B vitamins. Based on the timing of cobalt insertion and oxygen requirements, cobalamin can only be synthesized *de novo* by prokaryotes via either aerobic or anaerobic pathways (21). Specifically, precorrin-2 acts as the initial precursor shared by both the anaerobic and aerobic pathways of vitamin B_12_ biosynthesis. In the anaerobic route, cobalt chelation is mediated by the enzyme *CbiK*, whereas in the aerobic route, this process is performed by the *CobNST*. Research utilizing microbial cultivation methods has demonstrated that certain microbes are capable of synthesizing B vitamins, such as *Selenomonas ruminantium* (22), *Fibrobacter succinogenes* (23) and *Corynebacterium vitaeruminis* DSM −20,294 T (24).

Unlike humans and ruminants, the digestive system of chickens is relatively simple, comprising the stomach and the intestines, with the latter divided into the small intestine (duodenum, jejunum, ileum) and the large intestine (cecum, colorectum). Different intestinal segments of the chicken gut harbor distinct microbial communities (25), which result from differences in pH levels, physiological functions and retention time of digesta (26–28). However, previous research on vitamin biosynthesis has predominantly concentrated on the gut regions of humans and ruminants, leaving a gap in the comprehensive understanding of the entire gut microbiome in chickens. Therefore, whether the microbial biosynthesis of vitamins B and K_2_ is widespread in the chicken gut, and how different intestinal segments contribute to vitamin biosynthesis, remains insufficiently explored. Furthermore, there is evidence that pathogen infection (parasitic and bacterial infections) can disrupt the chicken gut microbiome (29–31), but the relationship between changes in microbial composition and vitamin production remains poorly understood. Therefore, gaining insight into the complex biosynthesis of vitamins B and K_2_ across the chicken gut is crucial for developing targeted strategies to regulate the microbiome and enhance vitamin production and utilization in chickens.

In this study, we systematically investigated the genomic potential for vitamins B and K_2_ biosynthesis within the chicken gut microbiome. A total of 195,517 non-redundant microbial genes and 3,063 high-quality genomes involved in vitamin biosynthetic pathways were identified. To capture both regional patterns and the effects of pathogen-induced perturbation, we analyzed raw metagenomic samples spanning multiple intestinal regions and infection conditions. Through these analyses, we revealed region-specific differences in the biosynthesis potential of key vitamins. Moreover, we observed significant shifts in microbial composition and vitamin biosynthesis capacity during bacterial (*Salmonella typhimurium*) and parasitic (*Eimeria tenella*) infections, underscoring a dynamic interaction between pathogen infection and vitamin biosynthesis. These findings could provide new insights for developing strategies to enhance the microbial synthesis of vitamins B and K_2_ in the chicken gut.

## 2 Materials and methods

### 2.1 Datasets

#### 2.1.1 Metagenome-assembled genome (MAG) dataset collection

A total of 25,825 MAGs were collected from multiple publicly available sources. Specifically, 12,339 MAGs were downloaded from the National Microbiology Data Center (NMDC)^1^ and 6,087 MAGs were retrieved from the Figshare repository^2^. In addition, 1,450 genomes from broiler chickens were obtained from the Zenodo repository,^3^ and 5,949 MAGs from laying hens were retrieved from the National Center for Biotechnology Information (NCBI) with the accession number PRJNA1099794.

#### 2.1.2 Raw metagenomic sample collection

From PRJNA417359, 135 metagenomic samples were collected from different regions of the chicken gut (duodenum, jejunum, ileum, cecum, and colorectum). From PRJNA1069780, 10 cecum samples were obtained, consisting of five control samples (NC) and five samples (ST) infected with *Salmonella typhimurium*. Additionally, from PRJNA943112, 12 chicken cecum samples were collected, which included four control samples (JC) and eight samples (IG) infected with *Eimeria tenella*. The IG group was further subdivided into a sensitive group (JS, *n* = 4) and a resistant group based on disease phenotypes (JR, *n* = 4). Metadata including sample type, gut segment, breed and experimental condition for all raw metagenomic samples are summarized in Supplementary Table 1.

### 2.2 Preprocessing and bioinformatic analysis

To ensure data quality, raw reads from all samples were processed for quality control using fastp v0.23.0 (32), applying the parameters ‘-q 20 -u 30 -n 5 -y -Y 30 -l 80 --trim_poly_g’. Host-derived sequences were subsequently eliminated through alignment against the reference genome (NCBI RefSeq assembly GCF_016699485.2) using Bowtie2 v2.5.0 (33) in end-to-end mapping mode (parameters: --end-to-end --mm --fast). The resulting non-host reads, designated as clean reads, were retained for downstream analyses.

The contamination and completeness of 25,825 genomes (isolated genomes and MAGs) were assessed using CheckM2 v1.0.1 (34). 17,901 microbial genomes met the inclusion criteria of <5% contamination, ≥50% completeness, and a quality score ≥50 (calculated as completeness − 5 × contamination). The genomes were taxonomically classified using the classify_wf workflow in GTDB-Tk v2.3.2 (35), based on the Genome Taxonomy Database (GTDB). Strain-level deduplication of the genomes was carried out using dRep v3.4.5 (36) with a 99% average nucleotide identity (ANI) threshold and the parameters ‘-pa 0.9 -sa 0.99 -nc 0.30 -cm larger --S_algorithm fastANI’, resulting in a dataset of 13,734 genomes. The 99% ANI threshold is a widely recognized standard for differentiating bacterial strains, providing an optimal balance between sensitivity and specificity in genome clustering (36, 37). Genomic comparisons employing identical genus-level taxonomic designations were subjected to average nucleotide identity (ANI) analysis through dRep v3.4.5 (parameters: -pa 0.9 -sa 0.95 -nc 0.30 -cm larger --S_algorithm fastANI). The ANI evaluation identified 2,675 species-level genome bins (SGBs) conforming to established species (>95% identity) and genus (>80% identity) thresholds. The phylogenetic tree generated via GTDB-Tk was visualized using iTOL v6.9.1.

### 2.3 Functional characterization of the microbial gene catalog related to vitamin biosynthesis

Open reading frames (ORFs) in the 13,734 genomes were predicted with Prodigal v2.6.3 (38) using the ‘-p single’ parameter. ORFs were clustered using the easy-cluster workflow in MMseqs2 (39), with the following parameter settings: ‘--split-mode 2 --cov-mode 2 -c 0.9 --min-seq-id 0.95 --cluster-mode 2 --cluster-reassign 1’. To perform functional annotation, the sequences were aligned with the Kyoto Encyclopedia of Genes and Genomes (KEGG) using DIAMOND v2.1.8.162 (40), with the parameters ‘--min-score 60 --query-cover 70 --max-target-seqs 5 --masking 1’. The alignment with the highest bit score was selected to represent the functional categorization of the ORFs.

Bowtie2 v2.5.0 was used to map the 20 million sequencing reads from 135 samples back to the non-redundant microbial gene catalog, and then the read counts in each sample were converted to transcripts per million (TPM). The relative abundances of KEGG orthologous groups (KOs) were derived from the abundance of their associated genes. In brief, for the taxonomic profiles, the total abundance for each phylum or genus was calculated by summing the abundances of all genes classified to that phylum or genus. The KO profiles were generated using the same method. KOs performing identical functions in the vitamin biosynthesis pathway were categorized into functional roles, and the abundances of these KOs were summed to represent the abundance of the respective functional role. The abundance of pathways associated with vitamins B and K_2_ biosynthesis was determined by summing the abundances of the KOs.

### 2.4 Phylogenetic, taxonomic, and functional characterization of 3,063 genomes

In addition, we assessed the quality of 25,825 genomes using CheckM2 v1.0.1. Genomes with ≥90% completeness and <5% contamination were retained. Strain-level de-redundancy of the genomes was carried out using dRep v3.4.5, applying the parameters ‘-pa 0.9 -sa 0.99 -nc 0.30 -cm larger --S_algorithm fastANI’, resulting in the identification of 5,411 genomes at a 99% ANI threshold. For functional annotation, BLASTP searches were conducted against the KEGG database using DIAMOND v2.1.8.162. We identified a set of essential functional roles that must be present in a genome for it to be classified as a vitamin producer capable of synthesizing vitamins B and K_2_ *de novo*. Using these essential functional roles, we identified 3,063 genomes predicted to synthesize vitamins B and K_2_ *de novo.* The phylogenetic tree was visualized using iTOL v6.9.1. A correlation network was created based on the biosynthetic capabilities of vitamins B and K_2_ in each genome. We conducted a comparative genomic analysis of 289 genomes (CCGs) possessing complete cobalamin *de novo* biosynthesis and 1,611 genomes (SCGs) belonging to the genera *Ligilactobacillus*, *Faecalibacterium*, *Corynebacterium*, *Mediterraneibacter*, *Blautia*, and *Limosilactobacillus*.

### 2.5 Analysis of the effects of pathogen infection on the chicken gut microbiota

We conducted a reanalysis of the metagenomic data from Ma et al. (41) and Tang et al. (42) regarding the chicken cecal microbiome in response to pathogen infection (bacterial and parasitic infections). Then, we used 195,517 non-redundant microbial genes as a gene database to assign metagenomic samples across all groups using Bowtie2 v2.5.0. Gene abundances were determined using TPM, and the differential analysis was conducted using the Wilcoxon rank-sum test.

### 2.6 Statistical analyses and visualization

All statistical analyses were conducted using R version 4.2.2. Alpha diversity was assessed using the abundance data from both taxonomic and functional gene features. Beta diversity was assessed using Bray-Curtis distance, and group differences were tested for statistical significance with permutational multivariate analysis of variance (PERMANOVA). The Wilcoxon rank-sum test was employed to examine significant variations in diversity indices. To control for multiple testing, false discovery rate (FDR) correction was applied to both PERMANOVA and Wilcoxon rank-sum tests used for pairwise comparisons.

Rarefaction curve was produced using the R package ‘vegan’ v2.6.4. Chord diagrams were visualized using the R package ‘circlize’ v2.8.0. Network graph was visualized using the software Gephi v0.10.1. Heatmap was visualized using R package ‘ComplexHeatmap’ v2.15.4. All other visualizations were generated using R package ‘ggplot2’ v4.2.3. A detailed reproducibility checklist listing software versions and key parameters is provided in Supplementary Table 2.

## 3 Results

### 3.1 Collection of genomes and gene catalog related to the gut microbiome of chicken

To obtain a comprehensive catalog of genomes and genes from the chicken gut microbiome, a total of 25,764 original MAGs and 61 isolated genomes were retrieved. Following quality assessment (≥50% completeness, <5% contamination, completeness – 5 × contamination ≥50) and redundancy removal at the 99% ANI level, we obtained 13,734 genomes that met or exceeded the quality standards for subsequent analysis. These genomes varied in size from 2.0 to 7.3 Mbp, with an average size of 2.1 Mbp. The average genome completeness was 85.7%, with an average contamination rate of 1.6%. Among these, 5,407 genomes (39.4%) were near-complete, exhibiting completeness ≥90% and contamination <5%. The average N50 and N90 lengths of the genomes were 38.1 Kbp and 11.5 Kbp, respectively (Supplementary Table 3).

**Figure 1:**
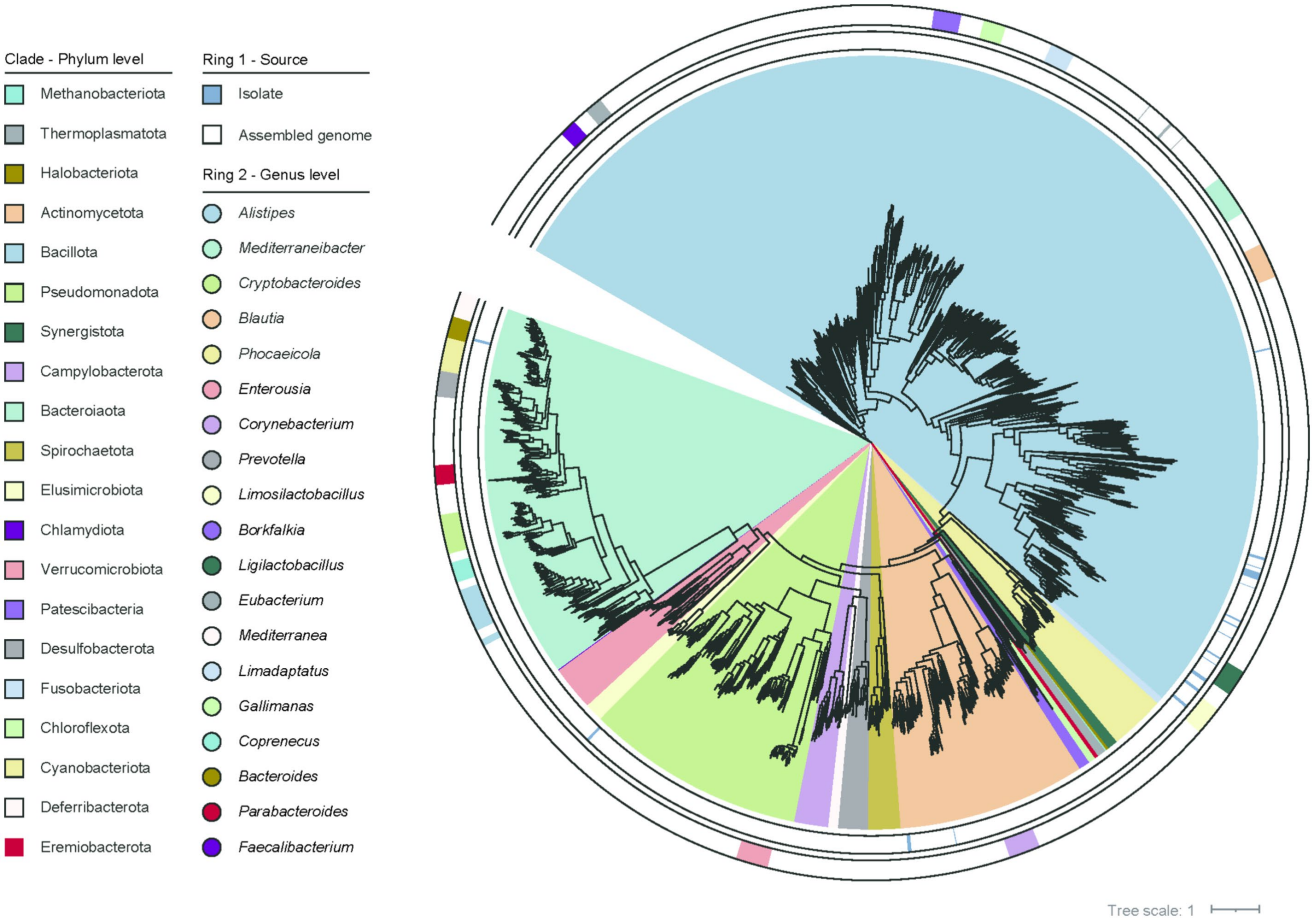
The phylogenetic relationships among the 2,675 genomes are illustrated. The first outer ring indicates the origin of the genomes and the second outer ring represents the genus-level classification of the genomes.

Next, we carried out a genome-wide clustering analysis to characterize the taxonomic composition of the chicken gut microbiome. This analysis resulted in a total of 2,675 SGBs based on a 95% ANI threshold at species boundaries. Based on the GTDB, 2,675 species were classified into 23 phyla, 35 classes, 88 orders, 204 families, and 773 genera. At the phylum level, the species were dominated by four bacterial phyla including *Bacillota* (54.7% of all species), *Bacteroidota* (16.0%), *Pseudomonadota* (9.5%), *Actinomycetota* (8.1%), followed by *Cyanobacteriota* (2.3%), *Verrucomicrobiota* (1.8%) and others. At the lower taxonomic levels, dominant taxa included *Oscillospirales* (15.1%; mainly consisting of *Faecalibacterium* and *Scatomorpha*), *Bacteroidales* (14.4%; mainly consisting of *Alistipes* and *Cryptobacteroides*), *Lachnospirales* (11.5%; mainly consisting of *Mediterraneibacter*), *Christensenellales* (9.4%; mainly consisting of *Borkfalkia, Limadaptatus* and *Gallimonas*) and *Lactobacillales* (5.7%; mainly consisting of *Limosilactobacillus* and *Ligilactobacillus*). These represented major clades of the chicken gut microbiota (Figure 1).

### 3.2 Abundance of vitamins B and K_2_ biosynthesis genes in the chicken gut microbiome

**Figure 2:**
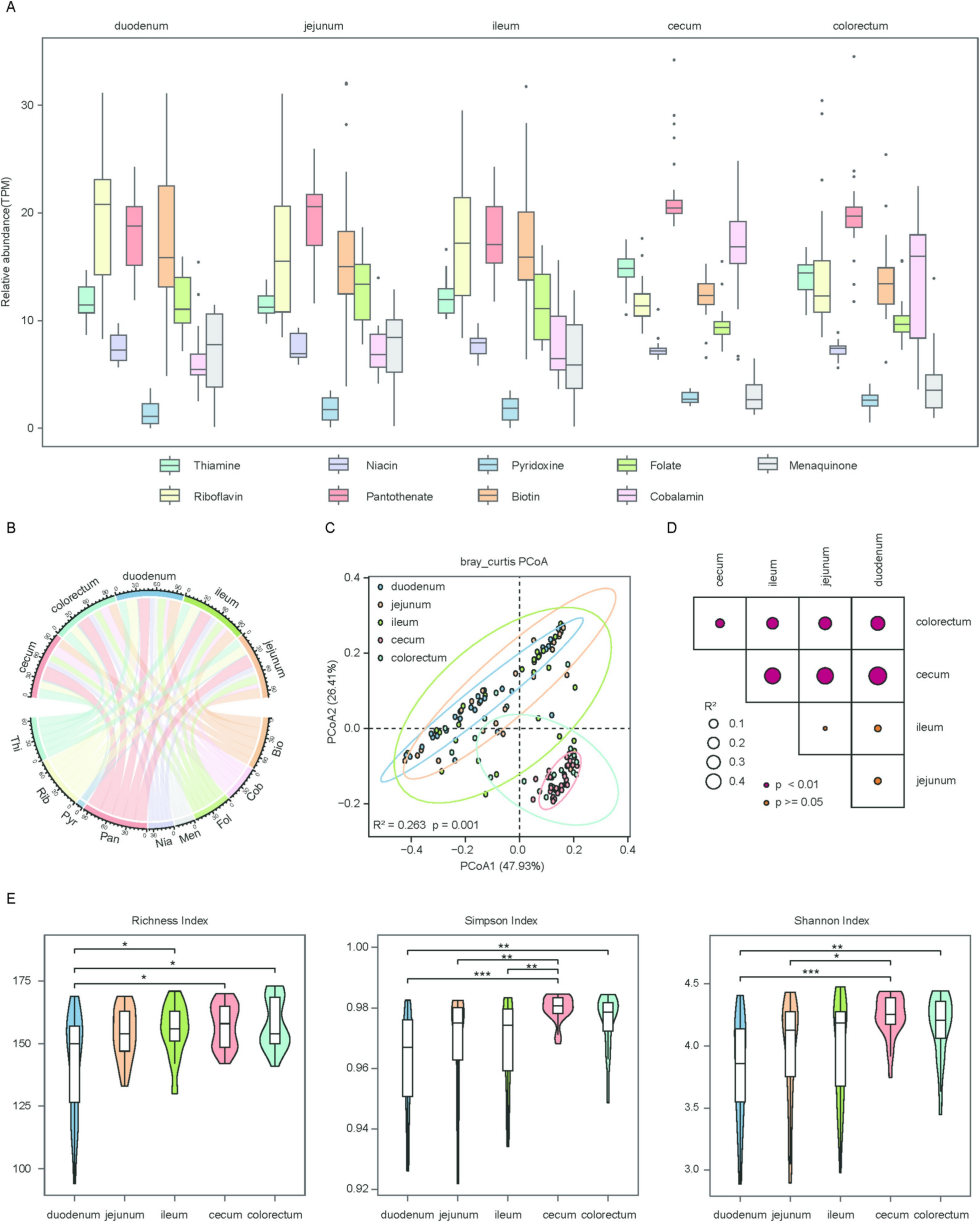
Regional distribution and diversity of microbial genes associated with vitamin biosynthesis in the chicken gut. **(A)** Box plots showing the overall abundance of microbial genes involved in the biosynthesis of vitamins B and K_2_ across five gut regions. Horizontal lines represent the medians, while the whiskers indicate the lowest and highest points within 1.5 × the interquartile ranges into the lower and upper quartiles. **(B)** Chord diagram illustrating the distribution of vitamin biosynthesis genes across different gut regions. Each gut region and each vitamin are represented by a different color. Vitamins B and K_2_ are abbreviated as: thiamine (Thi), riboflavin (Rib), niacin (Nia), pantothenate (Pan), pyridoxine (Pyr), biotin (Bio), folate (Fol), cobalamin (Cob), menaquinone (Men). **(C)** Scatter plot illustrating beta diversity, reflecting compositional changes in microbial genes associated with vitamins B and K_2_ biosynthesis. Samples are positioned along the first and second principal coordinates (PCoA1 and PCoA2), with the associated percentages of explained variance. Ellipsoids indicate the 95% confidence intervals for each group. **(D)** Results of the PERMANOVA analysis for pairwise gene group comparisons are shown. *p* value was determined using the adonis test with 1,000 permutations conducted in R. The color of points corresponds to the size of *p* value, and larger points reflect larger *R*^2^ values. **(E)** Box plots showing the richness (left panel), Simpson (medium panel) and Shannon (right panel) index of microbial genes involved in vitamins B and K_2_ biosynthesis. Significance levels were determined using the Wilcoxon rank-sum test: *p < 0.05; **p < 0.01; ***p < 0.001.

To elucidate the regional variations in microbe-mediated vitamin biosynthesis, we annotated the functions of the gene catalog. Approximately 60.2% of protein-coding genes could be mapped to the KEGG database, of which 195,517 genes and 192 KOs were identified as involved in the *de novo* biosynthesis of vitamins B and K_2_ (Supplementary Table 4). We first assessed the gene abundance involved in the biosynthetic pathways of vitamins B and K_2_. The biosynthesis of several vitamins exhibited distinct regional specialization along the chicken gut. Riboflavin (B_2_) and biotin (B_7_) biosynthesis were more abundant in the small intestine. Cobalamin (B_12_) biosynthesis was primarily observed in the large intestine. Pantothenate (B_5_) biosynthesis showed high abundance across all intestinal segments. In contrast, pyridoxine (B_6_) biosynthesis had the lowest abundance in all regions (Figures 2A,B). Additionally, we observed that the pathway abundance for the biosynthesis of vitamins B and K_2_ exhibited high regional heterogeneity throughout the whole gut segments. This was illustrated by PCoA, which showed that the visualization of the first two principal coordinate axes explained 74% of the total variation, and there was a clear separation among the different groups (*R*^2^ = 0.263, *p* = 0.001) (Figure 2C). The findings were further validated by the results of the PERMANOVA analysis (Figure 2D). To assess differences in the richness and evenness of biosynthetic pathway abundance among all groups, alpha diversity was calculated using the Richness, Shannon, and Simpson indices. Fisher’s method was used to combine *p*-values derived from Wilcoxon rank-sum tests for comparative analysis. The results showed that the duodenum had the lowest richness, whereas the cecum displayed the highest richness. Statistical comparisons revealed significant differences between the large and small intestine (Figure 2E). These findings highlight significant regional variation in the pathway abundance within the chicken gut.

To gain deeper insight into the biosynthesis of these vitamins, we examined the alternative biosynthetic pathways in the chicken gut microbiome (Supplementary Figures 1–3). The thiamine biosynthesis pathway consists of two branches, with the *thiI* branch being the predominant one observed in the chicken gut microbiome (Supplementary Figure 1A). The *ribD1* branch of riboflavin biosynthesis was more prevalent than the *RIB7* branch (Supplementary Figure 3C). The *NMNAT* branch of the niacin biosynthesis pathway was commonly observed in the chicken gut microbiome (Supplementary Figure 1C). The pyridoxine biosynthetic pathway comprises two branches, with the majority of genes in the longer branch exhibiting greater relative abundance in the large intestine. However, *pdxH*, which restricts the final step of the pathway, was relatively scarce (Supplementary Figure 3B). For biotin, four biosynthetic clades were present in the chicken gut microbiome, with the *FabG* branch being the most prevalent (Supplementary Figure 2A). Menaquinone biosynthesis involves two pathways, *Meq* and *Men*. Notably, most genes associated with the *Meq* pathway were more abundant in the large intestine, whereas those of the Men pathway were primarily enriched in the small intestine. However, the absence of two key KOs (K10106 and K05357) limited the *Meq* biosynthetic pathway (Supplementary Figure 2C).

### 3.3 Phylogenetic origin of vitamin biosynthetic genes

**Figure 3:**
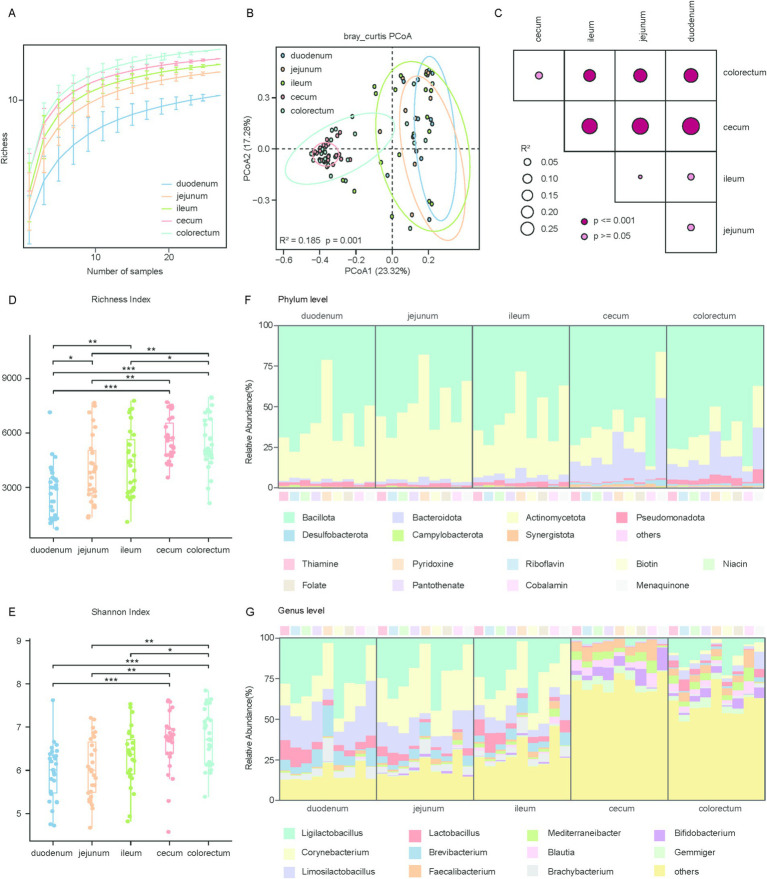
Phylogenetic and diversity analysis of microbial genomes involved in vitamin biosynthesis across gut regions. **(A)** Rarefaction analysis showing the richness of microbial genomes associated with vitamins B and K_2_ biosynthesis across five gut regions: duodenum, jejunum, ileum, cecum, and colorectum. The x-axis represents the number of samples randomly subsampled, and the y-axis indicates the observed richness. **(B)** Scatter plot illustrating beta diversity, reflecting compositional changes in microbial genomes associated with vitamins B and K_2_ biosynthesis. Samples are positioned along the first and second principal coordinates (PCoA1 and PCoA2), with the associated percentages of explained variance. Ellipsoids indicate the 95% confidence intervals for each group. **(C)** Results of the PERMANOVA analysis for pairwise genomes group comparisons. *p* values are determined using the adonis test with 1,000 permutations conducted in R. The color of points corresponds to the size of *p* value, and larger points reflect larger *R*^2^ values. **(D,E)** Box plots display the richness **(D)** and Shannon **(E)** index of microbial genomes involved in vitamins B and K_2_ biosynthesis. Significance levels were determined using the Wilcoxon rank-sum test: *p < 0.05; **p < 0.01; ***p < 0.001. **(F)** Phylogenetic distribution of vitamins B and K_2_ biosynthetic genes across the five gut regions at the phylum level. **(G)** Phylogenetic distribution of vitamins B and K_2_ biosynthetic genes across the five gut regions at the genus level.

In this study, we further investigated the spatial variations in species composition. Rarefaction curve analysis showed that the cumulative sequencing data had reached saturation, indicating that the sequencing effort had adequately covered the microbial genome diversity of chicken gut (Figure 3A). To explore the species composition in different segments of the chicken gut, we analyzed the relationships within and between all groups using alpha and beta diversity indices. PCoA based on Bray-Curtis distances was performed to assess the differences in species composition across various gut segments. The visualization of the first two principal coordinate axes explained 41% of the total variation (*R*^2^ = 0.185, *p* = 0.001) (Figure 3B). The results showed significant differences in overall species composition among the different segments of the chicken gut, which was further corroborated by the PERMANOVA (Figure 3C). To assess differences in species richness and evenness among the groups, we calculated the alpha diversity for each sample using two indices: the Shannon and Simpson. We then performed comparative analyses using the Fisher method combined with *p*-values from the Wilcoxon rank-sum test. The findings revealed that the species richness in the large intestine was substantially higher than in the small intestine (Figures 3D,E).

To understand the mechanisms underlying microbe-mediated vitamin biosynthesis, we performed a classification analysis of the genes involved in vitamin biosynthesis. Our analysis revealed that genes responsible for the biosynthesis of vitamins B and K_2_ were primarily affiliated with the phylum *Bacillota* (average relative abundance 63.29 ± 27.16% in all samples), followed by *Actinomycetota* (26.66 ± 29.42%) and Bacteroidota (6.31 ± 8.36%), This distribution differs from that observed in ruminants, whose vitamin-producing microbiota are typically dominated by *Bacteroidota* and *Firmicutes* (Figure 3F; Supplementary Figure 4A). Among these phyla, *Bacteroidota* showed a significant increase in relative abundance in the large intestine compared to the small intestine (Supplementary Figures 4B–D). It is noteworthy that biosynthetic genes for thiamine, riboflavin, niacin, pantothenate, biotin, folate, and cobalamin were predominantly derived from *Bacillota*, making up over 50% of the total, with cobalamin accounting for as much as 72.44%. Conversely, for menaquinone, 19.10% of the biosynthetic genes were assigned to the phylum *Bacteroidota*, a notably higher proportion compared to other vitamins (Supplementary Table 5).

The predominant genera associated with the biosynthetic genes for vitamins B and K_2_ were *Ligilactobacillus* (average relative abundance 19.62 ± 25.41% in all samples), *Corynebacterium* (12.71 ± 18.90%), *Limosilactobacillus* (9.02 ± 14.85%), *Lactobacillus* (4.01 ± 7.43%), *Brevibacterium* (3.54 ± 7.90%) and *Faecalibacterium* (3.41 ± 5.27%) (Figure 3G). Among these, *Ligilactobacillus*, *Corynebacterium*, *Limosilactobacillus* and *Lactobacillus* exhibited significantly higher abundance in the small intestine than in the large intestine. In contrast, *Faecalibacterium* was the only genus that showed significantly higher abundance in the large intestine compared to the small intestine (Supplementary Figures 4E–G). The taxonomic composition of vitamin biosynthesis genes in the large intestine was highly diverse, whereas in the small intestine, most biosynthetic genes were derived from *Ligilactobacillus* and *Corynebacterium,* except for Menaquinone, which was mainly synthesized by *Corynebacterium* and *Limosilactobacillus*. In summary, the dominant microbial communities involved in the biosynthesis of vitamins B and K_2_ differ throughout the chicken gut. Furthermore, distinct microbial taxa were associated with the biosynthesis of different vitamins within the chicken gut microbiome.

### 3.4 Collection of genomes for *de novo* vitamin biosynthesis

**Figure 4:**
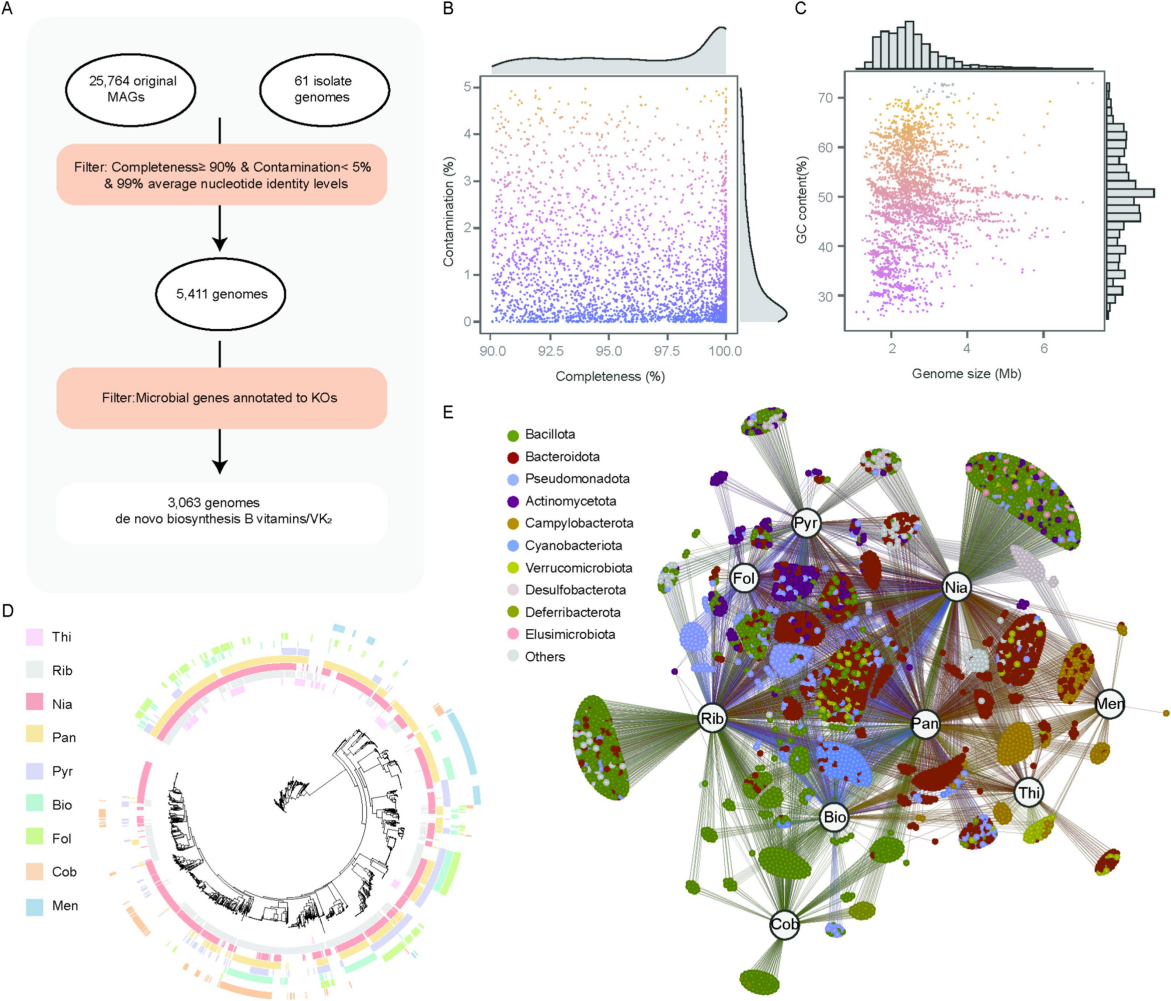
3,063 genomes identified as capable of synthesizing vitamins B and K_2._ **(A)** Workflow of identifying genomes capable of synthesizing vitamins B and K_2_. **(B,C)** Genomic statistics for 3,063 genomes. **(D)** Maximum-likelihood tree of 3,063 genomes, with clades color-coded based on the genome source. The outer-layer heatmaps indicate whether the corresponding genome possesses vitamin biosynthesis capabilities (colored) or lacks them (blank). **(E)** Correlation network of vitamins and genomes, each node represents a microbial genome, and its color corresponds to its assigned phylum. Larger labeled nodes represent specific vitamins: thiamine (Thi), riboflavin (Rib), niacin (Nia), pantothenate (Pan), pyridoxine (Pyr), biotin (Bio), folate (Fol), cobalamin (Cob), and menaquinone (Men). Edges represent predicted associations between genomes and the biosynthesis of corresponding vitamins.

We investigated the genome-level biosynthetic potential of vitamins B and K_2_ in the chicken gut microbiome. A total of 3,063 genomes were predicted to be capable of synthesizing at least one vitamin B or vitamin K_2_ *de novo* (Figure 4A; Supplementary Table 6). The size of these 3,063 genomes ranged from 1.08 to 7.29 Mbp (Figure 4B), with GC content ranging from 25.22 to 73.00% (Figure 4C). Taxonomic assignment showed that 1,119 and 864 genomes were classified into the two most abundant phyla, *Bacillota* (36.53%) and *Bacteroidota* (28.21%), respectively, followed by *Pseudomonadota* (*n* = 260, 8.49%), *Actinomycetota* (*n* = 189, 6.17%), *Campylobacterota* (*n* = 181, 5.91%), *Cyanobacteriota* (*n* = 101, 3.30%).

After evaluating the vitamin biosynthesis potential across these genomes, we identified that 1,027 genomes were capable of synthesizing a single type of vitamin, while 1,785 genomes could synthesize between two and four vitamins. Remarkably, no genome was found to synthesize all eight vitamins *de novo*, with only five genomes capable of producing seven vitamins, all of which belong to the genus *Bacillus*. This observation indicates that the majority of microorganisms obtain their synthesized vitamins through interactions with other microbes. Specifically, 242 genomes were linked to thiamine biosynthesis, 1,467 to riboflavin, 2,071 to niacin, 1,632 to pantothenate, 754 to pyridoxine, 534 to biotin, 424 to folate, 289 to cobalamin, and 274 to menaquinone (Figure 4D). Furthermore, 289 cobalamin-producing genomes were primarily classified within the phylum *Bacillota* (87.9%), whereas 274 menaquinone-producing genomes belonged to the phylum *Campylobacterota* (62.4%). These *campylobacterota*-affiliated genomes were unable to synthesize thiamine, pyridoxine, folate, or cobalamin. Notably, all genomes from the genera *Bacteroidota*, Cor*ynebacterium, Megamonas* and *Prevotella* could synthesize three or more vitamins, suggesting their broad vitamin biosynthetic potential. Interestingly, we also observed that folate was mainly synthesized by *Bacteroidota*-affiliated genomes (41.98%) and *Pseudomonadota*-affiliated genomes (27.59%), whereas cobalamin was primarily synthesized by *Bacillota*-affiliated genomes (87.89%) (Figure 4E; Supplementary Table 7). However, the majority of *Bacteroidota*-associated genomes were documented to produce cobalamin and folate within the human gastrointestinal tract (9), and *Bacteroidota*-affiliated genomes in the gastrointestinal tract of ruminants are unable to synthesize folate and cobalamin (20). These findings suggest that there are differences in vitamin biosynthesis capabilities between the chicken gut microbiota and the gut microbiota of humans and ruminants.

### 3.5 Comparative genomics of cobalamin biosynthesis in the chicken gut microbiome

**Figure 5:**
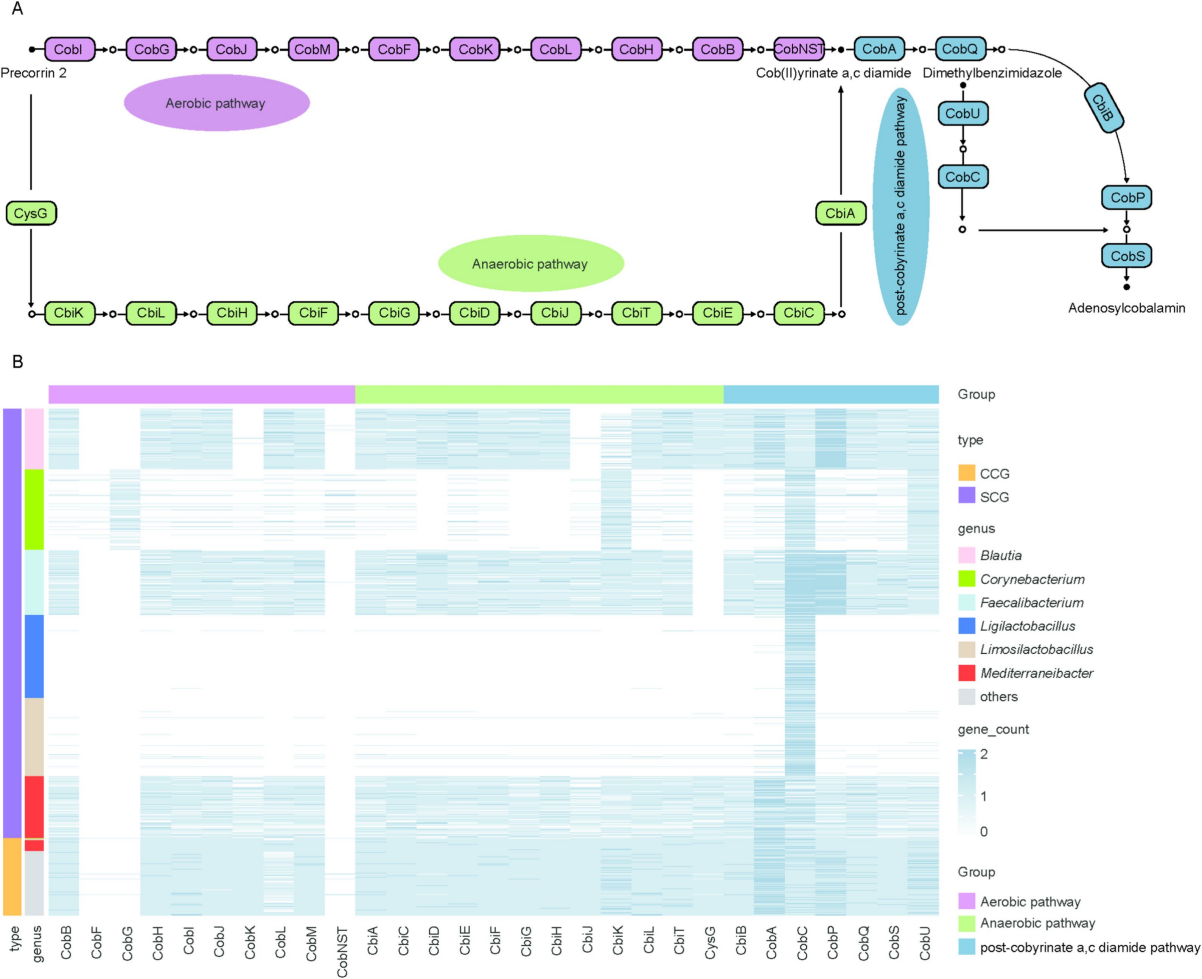
*De novo* biosynthesis pathway of cobalamin and distribution of functional roles across genera **(A)**. Diagram of the cobalamin *de novo* biosynthesis pathway. Rectangles represent functional roles and circles represent metabolites **(B)**. Each row represents an individual genome, and each column represents a gene involved in cobalamin biosynthesis. The color intensity reflects the number of genes per genome, with darker blue indicating higher numbers. Gene functions are categorized into three biosynthetic stages: the aerobic pathway (purple bar), the anaerobic pathway (green bar), and the post-cobyrinate a,c-diamide pathway (light blue bar). The first column of bar plots on the left represents the classification of genomes with complete cobalamin biosynthetic pathways (CCGs, orange) and those with partial pathways (SCGs, blue). The second column of bar plots on the left represents the taxonomic genera to which the genomes belong.

Cobalamin, an essential cofactor, is exclusively synthesized by prokaryotic organisms (43). Its microbial production occurs through two distinct biosynthetic routes, distinguished by the timing of cobalt incorporation and the dependence on molecular oxygen: the aerobic and anaerobic pathways. In the aerobic route, *precorrin 2* undergoes methylation to form *precorrin 3A* via the action of *cobI*, followed by cobalt chelation mediated by *cobNST*. Conversely, in the anaerobic pathway, cobalt is incorporated into *precorrin 2* early, yielding cobalt-sirohydrochlorin in the second step. Both pathways ultimately converge at the formation of cob(II)yrinate a,c diamide, which is subsequently transformed into the *cobamide* coenzyme (Figure 5A).

The *de novo* biosynthesis pathway of cobalamin was primarily associated with members of the genera *Ligilactobacillus, Faecalibacterium, Corynebacterium, Mediterraneibacter, Blautia, and Limosilactobacillus.* However, genomes belonging to these six genera were found to have incomplete gene sets for *de novo* cobalamin biosynthesis. This raises the question of which specific genes are absent in these genomes, thereby limiting their ability to synthesize cobalamin *de novo*.

To address this, we analyzed 1,611 genomes (SCGs) from these six genera, which contained partial genes associated with cobalamin biosynthesis, and compared them with 289 genomes (CCGs) that harbored complete genes for *de novo* cobalamin biosynthesis. Our analysis revealed that the majority of CCGs possessed all necessary genes for the anaerobic and post-cobyrinate a,c diamide pathways but lacked key genes (*CobF, CobG*, and *CobNST*) required for the aerobic pathway, indicating that cobalamin biosynthesis in the chicken gut microbiome predominantly occurs through the anaerobic pathway. This is consistent with observations in ruminants. Similarly, most SCGs lacked the essential genes *CobNST*, which is required for the aerobic pathway and catalyzes corrin ring synthesis by transforming *precorrin 2* into *precorrin 6A*. Additionally, over 90% of *Blautia*-associated genomes were deficient in five essential genes (*CobF*, *CobG*, *CobK*, *CobNST* and *CbiJ*). All *Mediterraneibacter*-affiliated genomes lacked *CobF*, *CobG* and *CobNST* genes and almost all *Faecalibacterium*-associated genomes also lost *CobF*, *CobG* and *CobNST* genes. Genomes from the remaining three genera were missing even more essential genes required for cobalamin biosynthesis (Figure 5B).

### 3.6 Effects of the pathogen infection on vitamin biosynthesis in the chicken gut microbiome

**Figure 6:**
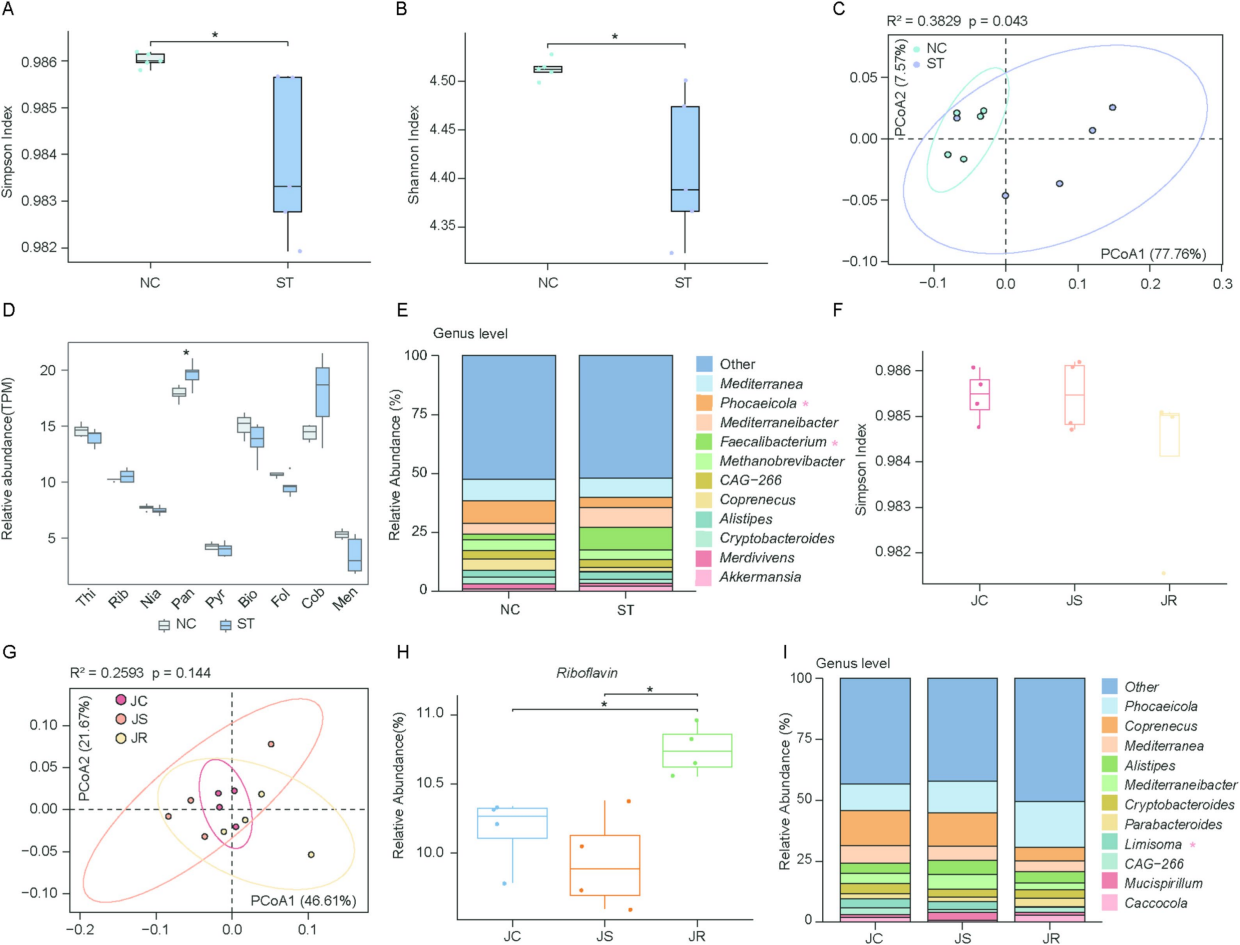
Comparison of vitamins B and K_2_ biosynthesis during pathogen infection. **(A,B)** Box plots showing the Simpson **(A)** and Shannon **(B)** indices of microbial genes involved in vitamins B and K_2_ biosynthesis in the normal control (NC) and *Salmonella typhimurium*-infected (ST) groups. Statistical significance was assessed using the Wilcoxon rank-sum test: *p < 0.05. **(C)** Scatter plot illustrating beta diversity, reflecting compositional changes in microbial genomes associated with vitamins B and K_2_ biosynthesis. Samples are positioned along the first and second principal coordinates (PCoA1 and PCoA2), with the associated percentages of explained variance. Ellipsoids indicate the 95% confidence intervals for each group. **(D)** Box plot showing the overall abundance of microbial genes associated with vitamins B and K_2_ biosynthesis between the NC and ST groups. Horizontal lines represent the medians, while the whiskers indicate the lowest and highest points within 1.5 × the interquartile ranges into the lower and upper quartiles, respectively. **(E)** Phylogenetic distribution of vitamins B and K_2_ biosynthetic genes between NC and ST groups at the genus level. **(F)** Box plots showing the Simpson index of vitamin biosynthetic genes among the uninfected control (JC), *Eimeria tenella*-sensitive (JS), and *E. tenella*-resistant (JR) groups. **(G)** Scatter plot illustrating beta diversity, reflecting compositional changes in microbial genomes associated with vitamins B and K_2_ biosynthesis. **(H)** Box plot showing the overall abundance of microbial genes associated with vitamins B and K_2_ biosynthesis across the three groups. **(I)** Phylogenetic distribution of vitamins B and K_2_ biosynthetic genes among three groups at the genus level.

The gut microbiota plays a central role in vitamin biosynthesis. However, how pathogen infections—particularly bacterial and parasitic—affect the composition and functional capacity of vitamin-producing microbiota remains poorly understood. Therefore, we reanalyzed metagenomic sequencing data from chickens infected with bacterial and parasitic pathogens. Moreover, we observed shifts in microbial composition and vitamin biosynthesis capacity during infections with (*Salmonella typhimurium*) and parasitic (*Eimeria tenella*), highlighting a dynamic interaction between the pathogen infection and vitamin biosynthesis. Using our vitamin biosynthesis gene dataset, we observed that the alpha diversity of genes involved in the vitamins B and K_2_ biosynthesis pathways in the ST group was markedly reduced compared to the NC group (Simpson: *p* = 0.01, Cohen’s d = 1.78, Shannon: *p* = 0.02, Cohen’s d = 1.92) (Figures 6A,B). This result was further corroborated by the PCoA, which showed a clear separation between the ST and NC groups (*R*^2^ = 0.383, *p* = 0.043) (Figure 6C). Genes involved in pantothenate biosynthesis were enriched in the ST group (*p* = 0.032) (Figure 6D). We then performed a taxonomic analysis to identify the microbial taxa contributing to vitamin biosynthesis. After infection with *Salmonella typhimurium*, substantial alterations were observed in the microbial communities of the genera *Phocaeicola* and *Faecalibacterium* (Figure 6E). For *Eimeria tenella* infections, we observed that neither alpha diversity nor beta diversity showed significant differences among the three groups (*R*^2^ = 0.2593, *p* = 0.144) (Figures 6F,G). Riboflavin biosynthesis gene abundance was significantly higher in the JR group than in the JC and JS groups (Figure 6H), suggesting that the enhanced riboflavin biosynthesis capacity of the gut microbiota in the JR group may be associated with its resistance. Taxonomic analysis revealed that the abundance of *Limisoma* significantly changed (Figure 6I).

## 4 Discussion

Vitamins, particularly B and K_2_, are synthesized by the microbiome and play critical roles in fundamental biological processes within both microbial communities and host cells. However, the biosynthesis of vitamins B and K_2_ within the chicken gut microbiome remains largely unexplored, hindering efforts to effectively modulate host–microbe interactions for improved stability and health. In this study, we conducted a metagenomic analysis and provided a comprehensive understanding of the biosynthesis of vitamins B and K_2_ in the chicken gut microbiome. These vitamins are essential for poultry growth, immune function and overall health. Our study involved the collection and characterization of 25,764 MAGs and 61 isolates, revealing a highly diverse microbial ecosystem capable of contributing significantly to the host’s nutrient supply.

One of the most significant findings from our analysis was the substantial regional variation in the biosynthesis of vitamins B and K_2_ within the chicken gut. Similar to observations in ruminants, vitamin biosynthesis differed significantly between the small and large intestines (20). For instance, the biosynthesis of riboflavin and biotin was more active in the small intestine. These vitamins are essential for energy metabolism and fatty acid synthesis, functions that closely align with the physiological roles of the small intestine (44–46). In contrast, the large intestine typically has a lower pH and more complex fermentation functions, favoring the synthesis of certain vitamins by anaerobic microbes, such as thiamine (20, 47). The cecum, in particular, exhibited the highest richness of vitamin biosynthetic genes. This may be attributed to its distinct physiological and ecological features, including prolonged digesta retention time, low oxygen levels, and a rich pool of fermentable substrates—conditions that promote the growth of obligate anaerobes and favor the *de novo* biosynthesis of complex cofactors such as menaquinone and cobalamin (26–28). These vitamins play essential roles in host coagulation, bone metabolism, and energy homeostasis, underscoring the functional importance of the cecal microbiota in supporting host nutritional needs (48–50). These region-specific patterns not only clarify the spatial functional organization of the chicken gut microbiota but also suggest new avenues for intervention. Targeted dietary strategies—such as region-specific prebiotics or the administration of probiotic strains adapted to cecal conditions—could be developed to enhance the colonization and activity of vitamin-producing microbes (7).

Our findings also highlighted the complex interplay between diverse microbial communities and the biosynthesis of vitamins B and K_2_. Microbial taxa such as *Bacillota* and *Actinomycetota* were major contributors, emphasizing their critical roles in nutrient synthesis and host health (51). Their widespread presence across different gut segments suggests a pivotal role in synthesizing essential vitamins—nutrients that are indispensable for host growth and immune function (52, 53). Interestingly, our study revealed a high degree of microbial auxotrophy, with only 8.2% genomes able to synthesize five or more vitamins. This suggests a highly interdependent microbial ecosystem where such interactions are crucial for the synthesis of complex molecules like vitamins (54, 55). Our findings have significant implications for poultry health management and dietary strategies. Understanding the vitamin biosynthesis landscape across the chicken gut allows for targeted intervention to enhance vitamin production. This approach not only supports the health and productivity of poultry but also reduces reliance on external vitamin supplementation, thereby decreasing costs and improving the sustainability of poultry farming.

Cobalamin, a unique and complex vitamin, is synthesized exclusively by certain bacteria and archaea (56). It serves as a cofactor in various essential metabolic pathways, including DNA synthesis, fatty acid metabolism, and amino acid biosynthesis (57). Our results indicated that cobalamin biosynthesis primarily occurs in the large intestine through an anaerobic pathway. The large intestine provides a favorable environment for anaerobic fermentation, characterized by slower transit times, higher microbial density, and the presence of substrates that support the growth of cobalamin-producing bacteria (58, 59). The slow passage of digesta through the large intestine extends the time available for microbial activity, which is likely crucial for the complex series of reactions required for cobalamin biosynthesis (60). The concentration of cobalamin synthesis in the large intestine has important implications for both microbial ecology and host nutrition. The interdependence among gut microbes, in which some bacteria produce cobalamin while others utilize it, may enhance the stability of the microbial community in this region (61, 62). This microbial cross-feeding, coupled with the large intestine’s unique environment, underscores the critical role of this gut segment in providing essential nutrients like cobalamin to both the host and other gut microbiota members. Interestingly, the localization of cobalamin synthesis in the large intestine of chicken contrasts with patterns observed in ruminants, where cobalamin production is more prevalent in the small intestine (20). This difference likely stems from variations in diet, gut anatomy, and microbial community structure between species.

We investigated how vitamin-related gut microbiota respond to bacterial and parasitic infections. Significant differences in alpha and beta diversity of vitamin B and K_2_ biosynthetic genes were observed between the ST group and the control group (NC), with reduced gene diversity in the ST group, indicating gut dysbiosis and impaired vitamins biosynthesis potential (63). Interestingly, pantothenate biosynthesis genes were enriched in the ST group, potentially reflecting a compensatory microbial response to infection-induced stress, as pantothenate supports energy metabolism and immune regulation (64). In contrast, parasitic infection did not significantly affect gut microbiota alpha or beta diversity. However, the parasite-resistant JR group showed a higher abundance of riboflavin synthesis genes, suggesting enhanced riboflavin production linked to infection resistance, consistent with previous findings (65). While these findings provide important insights into the functional dynamics of the chicken gut microbiota during infection, they are based solely on metagenomic data, which assesses gene presence and relative abundance but do not capture gene expression activity. Therefore, it remains unclear whether the observed changes in vitamin biosynthetic potential are driven by shifts in microbial composition, alterations in gene expression, or both. In conclusion, our findings suggest that different types of pathogens influence the composition of microbial taxa and the functional potential of the microbiota in synthesizing essential vitamins. The underlying mechanisms driving these microbial and functional shifts may include inflammation-induced suppression of beneficial microbes and competitive displacement by infection-tolerant taxa. For instance, *Salmonella Typhimurium* infection induces intestinal inflammation that increases luminal oxygen and nitrate levels, favoring facultative anaerobes and suppressing strict anaerobes such as *Faecalibacterium* (30, 66, 67). In parallel, pathogen-induced niche competition may lead to the decline of taxa such as *Phocaeicola*, potentially displaced by opportunistic species better adapted to inflammatory stress (68). These findings point to a dynamic interplay between host immune status, microbial composition, and metabolic function. However, a major limitation of this study lies in the relatively small sample sizes used for pathogen infection comparisons. Although our analysis revealed trends in vitamin biosynthesis pathways and associated taxa, the limited sample size may affect the generalizability of our findings. Future studies with larger and more diverse cohorts are needed to validate these observations, enhance statistical robustness, and improve the applicability of our findings across different poultry populations and farming environments.

Although the study offers comprehensive computational insights into the biosynthetic potential of vitamins B and K_2_ in the chicken gut microbiome, and highlights a robust and adaptable microbial system capable of synthesizing essential vitamins under varying physiological and environmental conditions, it is important to acknowledge that the conclusions are based solely on metagenomic predictions and lack direct experimental validation. This limitation restricts our ability to distinguish whether the observed functional differences are due to changes in microbial community composition, transcriptional regulation, or both. Therefore, future studies should incorporate multi-omics approaches to verify the functional relevance of the predicted biosynthetic genes. For instance, quantitative PCR (qPCR) or RNA-Seq could be used to assess gene expression levels of key biosynthetic enzymes, while targeted or untargeted metabolomics could directly measure vitamin concentrations across gut regions and under infection conditions. These validations would provide stronger evidence for the functional contributions of gut microbes to vitamin biosynthesis in chickens, further supporting microbiome-based nutritional interventions in poultry.

## 5 Conclusion

In this study, we collected 25,825 microbial genomes to investigate microbe-mediated biosynthesis of vitamins B and K_2_ in the chicken gut. We found significant regional variations in gene abundance across the chicken gut. Genes involved in vitamin biosynthesis were predominantly affiliated with the phyla *Bacillota, Actinomycetota*, and *Bacteroidota*. Using this gut vitamin gene catalog, we identified 3,063 genomes from multiple phyla predicted to synthesize at least one vitamin B or vitamin K_2_ *de novo*, with several critical trophic metabolisms interacting with vitamin biosynthesis. However, only 8.2% of genomes were capable of synthesizing five or more vitamins, while approximately half were limited to producing just one vitamin, suggesting that the majority of microbes in the chicken gut microbiome are auxotrophic. Notably, we observed that only a limited number of microbial genomes contained the complete pathway for *de novo* cobalamin biosynthesis. In addition, we observed significant shifts in microbial composition and vitamin biosynthesis during bacterial (*Salmonella typhimurium*) and parasitic (*Eimeria tenella*) infections, suggesting that infections may alter the microbiome’s ability to produce essential vitamins, potentially influencing the health of the host. In conclusion, our findings deepen the understanding of gut microbiota-mediated vitamin biosynthesis in chickens and highlight the dynamic interplay among microbiome composition, pathogen infection, and vitamin production. These insights provide a foundation for targeted regulation of microbial communities to enhance the biosynthesis of essential vitamins in poultry.

## Data Availability

The original contributions presented in the study are included in the article/Supplementary material, further inquiries can be directed to the corresponding author.

## Footnotes

^1^ https://nmdc.cn/icrggc/

^2^ http://doi.org/10.6084/m9.figshare.24681096.v1

^3^ https://doi.org/10.5281/zenodo.14432035
