# Supplementary figures and images for: Insights from metagenomics on microbial biosynthesis of vitamins B and K2 in chicken gut microbiota
Source: Front Vet Sci. 2025 Aug 14;12:1646825. doi: 10.3389/fvets.2025.1646825 (PMC12393000; doi:10.3389/fvets.2025.1646825)

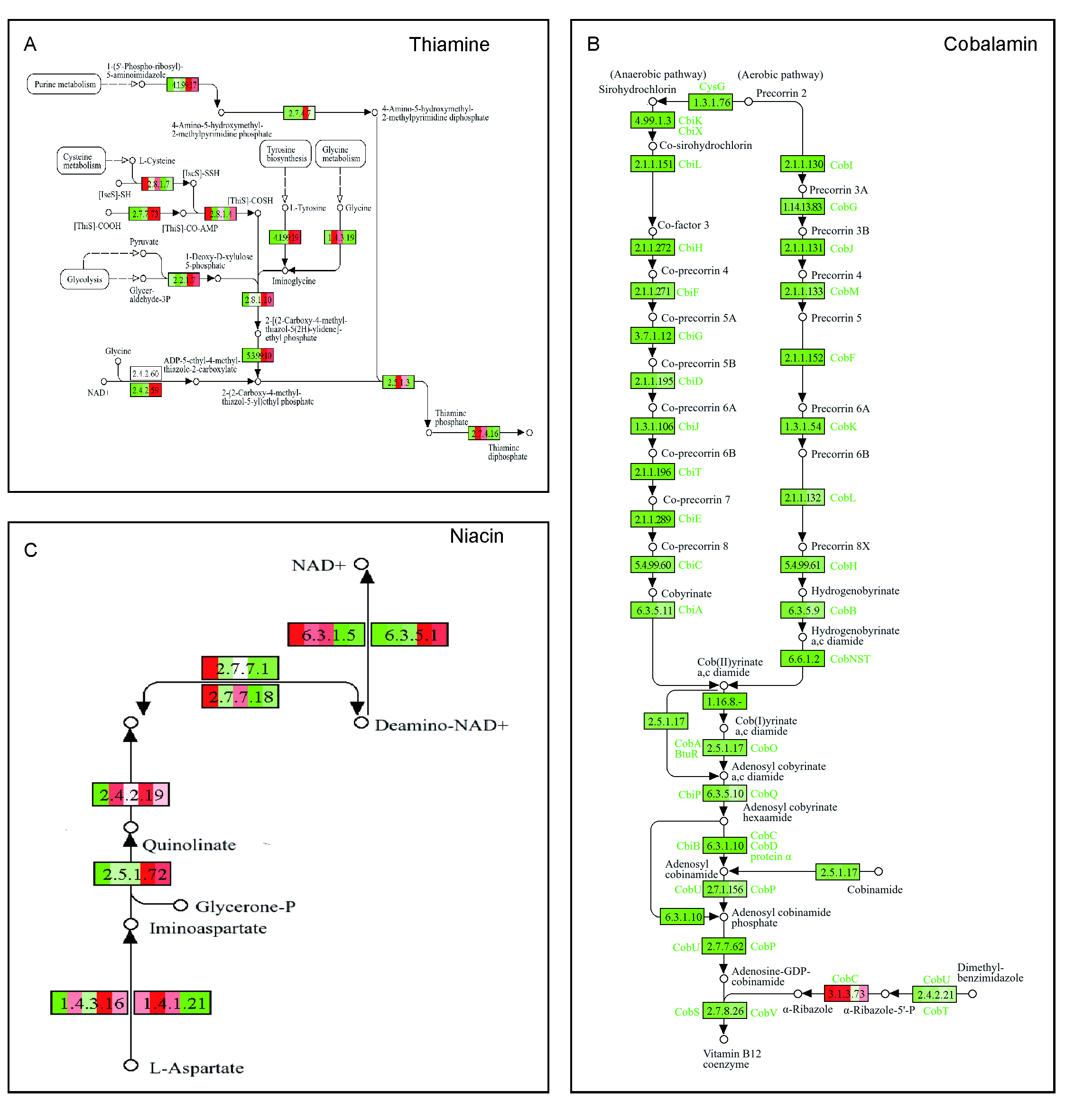

Supplement: Supplementary file 1 [file Image_1.tiff]

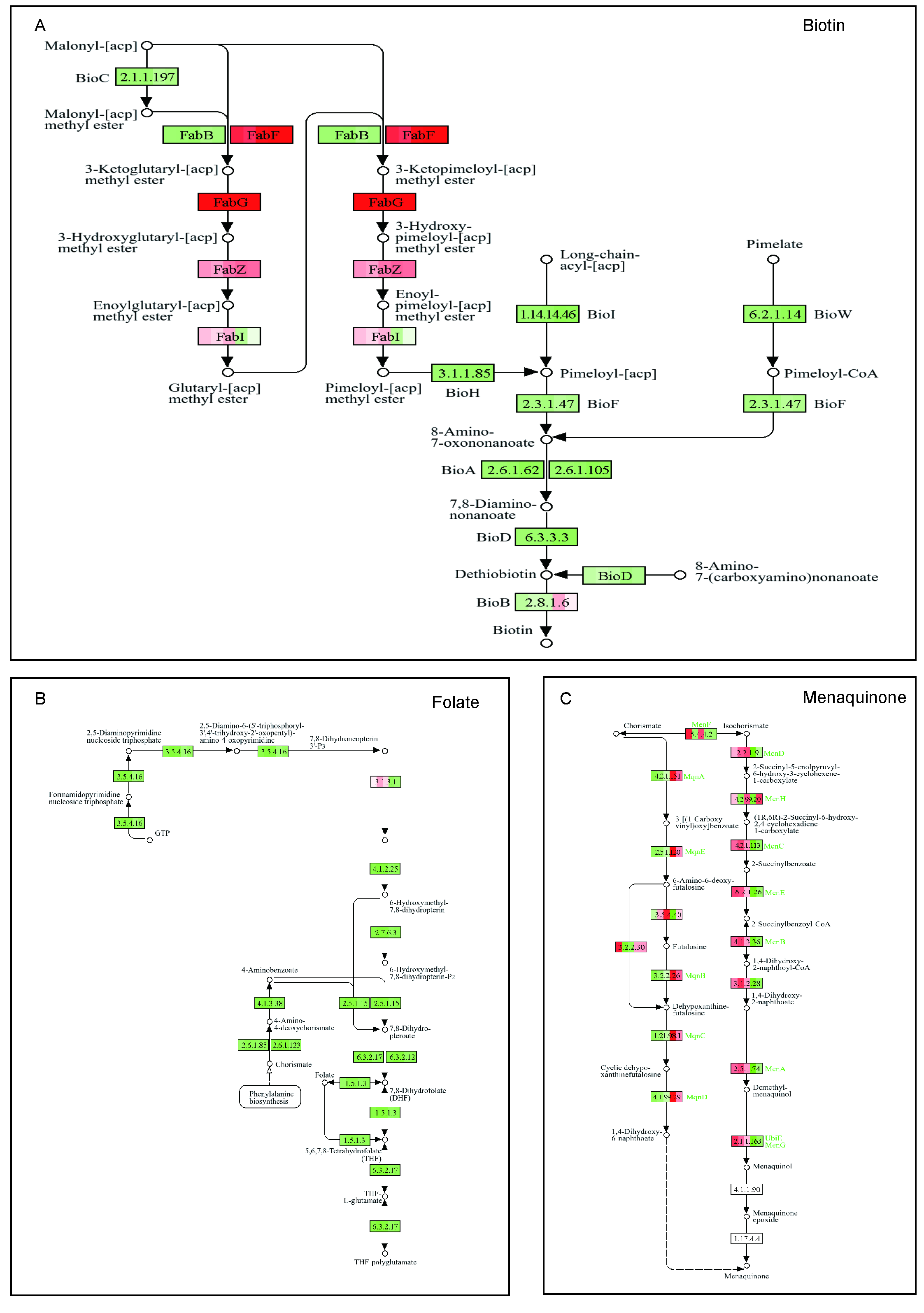

Supplement: Supplementary file 2 [file Image_2.tiff]

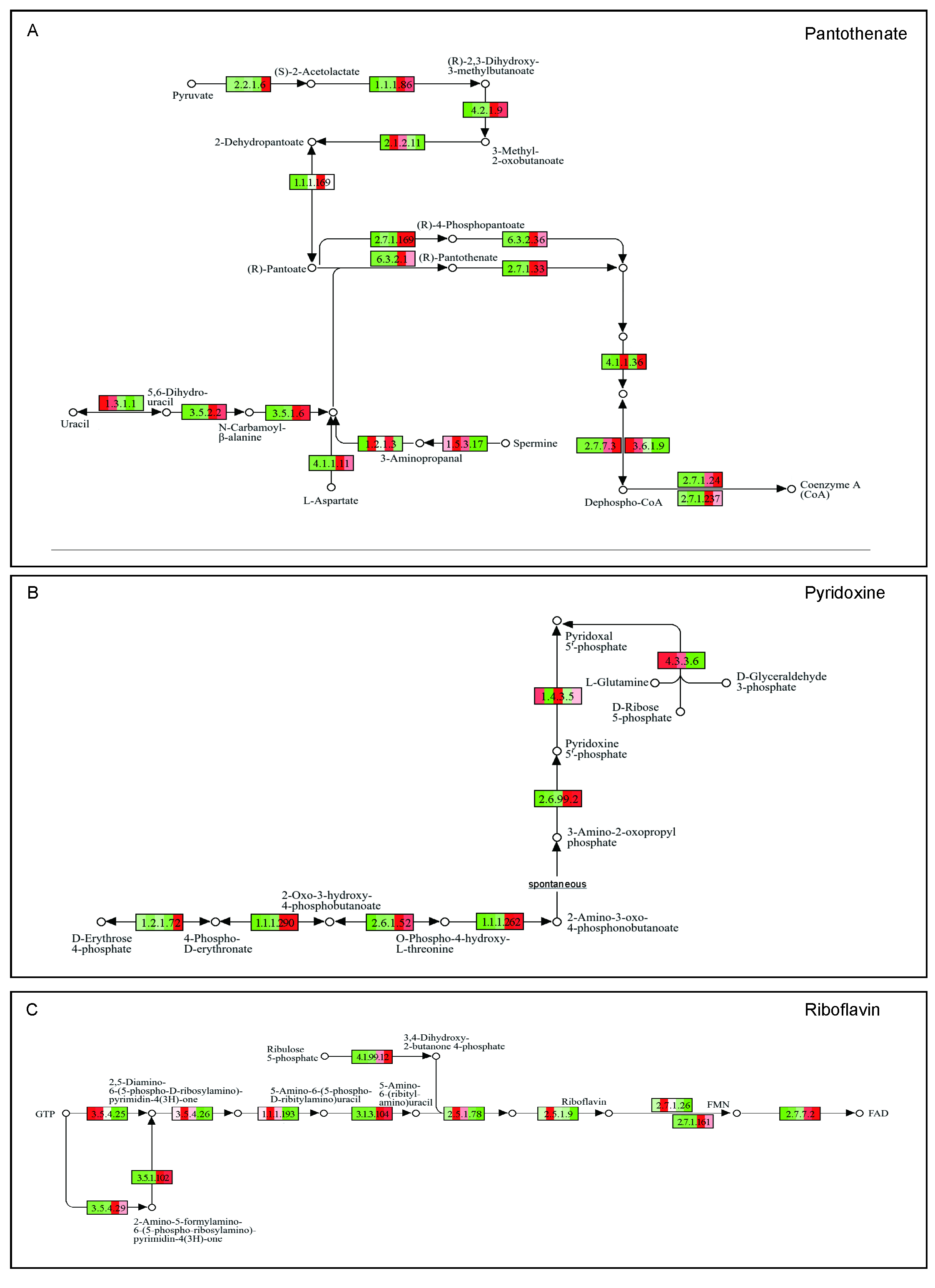

Supplement: Supplementary file 3 [file Image_3.tiff]

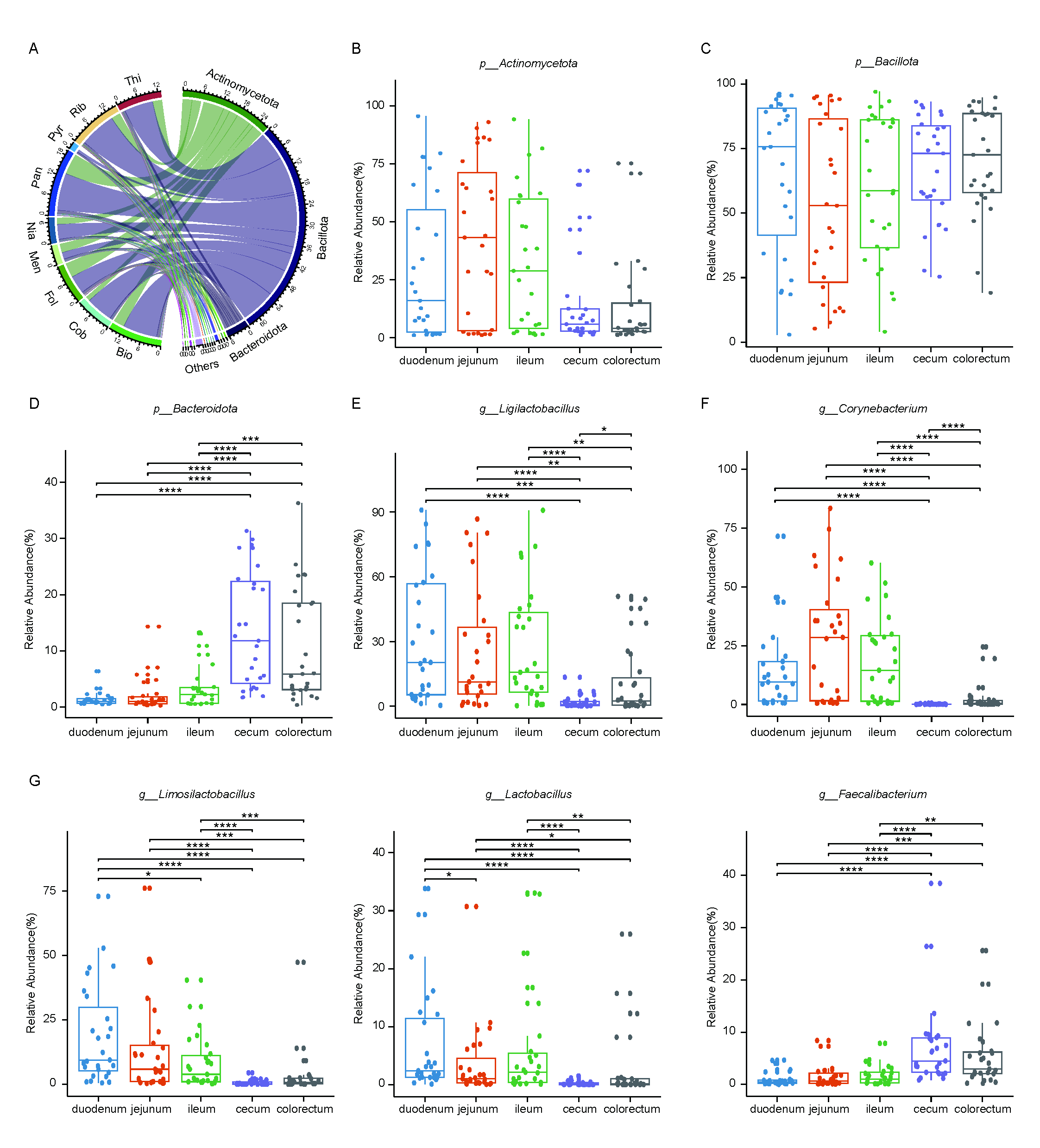

Supplement: Supplementary file 4 [file Image_4.tiff]
